# Prevalence and Risk Factors for Undiagnosed Glucose Intolerance Status in Apparently Healthy Young Adults Aged <40 Years: The Korean National Health and Nutrition Examination Survey 2014–2017

**DOI:** 10.3390/ijerph16132393

**Published:** 2019-07-05

**Authors:** Young Sang Lyu, Sang Yong Kim, Hak Yeon Bae, Jin Hwa Kim

**Affiliations:** Department of Endocrinology and Metabolism, Chosun University Hospital, Gwangju 61453, Korea

**Keywords:** undiagnosed diabetes, undiagnosed prediabetes, young adults

## Abstract

*Background:* Early-onset diabetes results in longer lifetime hyperglycemic exposure that consequently leads to earlier chronic diabetes complications and premature death. The aim of this study was to quantify the prevalence and risk factors of undiagnosed diabetes and undiagnosed prediabetes in apparently healthy young adults aged <40 years. *Methods:* This study used data from the Korean National Health and Nutrition Examination Survey, a cross-sectional, nationally representative survey conducted by the Korean Ministry of Health and Welfare from 2014 to 2017. A total of 4442 apparently healthy young adults enrolled in this study. Multivariate logistic regression analyses were conducted separately to evaluate associated risk factors with undiagnosed diabetes and undiagnosed prediabetes in groups stratified by sex. *Results:* The prevalence of undiagnosed diabetes and undiagnosed prediabetes was 1.2% and 25.0%, respectively. Obesity (body mass index ≥ 30.0 kg/m^2^) was a significant risk factor of undiagnosed diabetes regardless of sex (men, odds ratio (OR): 9.808, 95% confidence interval (CI): 1.619–59.412; women, OR: 7.719, 95% CI: 1.332–44.747). Family history of diabetes was significantly associated with undiagnosed diabetes (OR: 3.407, 95% CI: 1.224–9.481) in women only. Increased age, obesity status, and family history of diabetes were significant risk factors for undiagnosed prediabetes. Alcohol consumption was found to be negatively associated with undiagnosed prediabetes in women. *Conclusions:* Increased attention and implementation of precise strategies for identifying young adults at high risk for undiagnosed diabetes would allow for increased wellbeing as well as reduced healthcare burdens associated with diabetes.

## 1. Introduction

Increased incidence of diabetes has resulted in an increased prevalence of diabetes in young adults. Data from the International Diabetes Federation showed a dramatic global increase in young adults aged 20–39 years with type 2 diabetes from 23 million cases in the year 2000 to 63 million in 2013 [1]. 

Early-onset diabetes results in an overall longer lifetime hyperglycemic exposure, which can consequently lead to earlier chronic diabetes complications and an increased risk of later life cardiovascular disease (CVD) development [2,3]. Age at first diabetes diagnosis is also associated with loss of life years and is higher for those diagnosed with diabetes at a younger age (20.0 life-years lost for those diagnosed at 20 years, 10.6 life-years lost for those diagnosed at 40 years, and 4.5 life-years lost for those diagnosed at 60 years) [4]. 

The young adult population has been commonly undiagnosed despite a recognized need for earlier diabetes identification [5,6]. Identification of at-risk young adults may allow for earlier precision interventions used to manage or prevent diabetes that would lead to improved quality of life. Prediabetes places an individual at high risk for diabetes [7]. Therefore, identifying individuals who are at risk of developing prediabetes is also critical for diabetes prevention. The risk of diabetes is increased with obesity, increasing age, sedentary lifestyle, and unhealthy diet [8].

However, despite the emphasis on early, selective screening, few studies have reported the risk factors associated with undiagnosed diabetes in young adults. To the best of our knowledge, there are no large population-based reports examining undiagnosed prediabetes limited only to apparently healthy young adults. The objective of this study was to evaluate the prevalence as well as associated risk factors of undiagnosed diabetes and prediabetes in apparently healthy young adults aged <40 years. 

## 2. Materials and Methods

### 2.1. Study Population

This study used data from the Korean National Health and Nutrition Examination Survey (KNHANES) conducted from 2014 to 2017 by the Korean Ministry of Health and Welfare. The participants were recruited using a stratified, multistage, and clustered probability sampling design. Sampling units were defined based on data from household registries, including geographic area, sex, and age groups. This survey was a cross-sectional, nationally representative study of noninstitutionalized civilians. The KNHANES consisted of a health interview survey, nutrition survey, and health examination survey conducted by trained investigators. All survey participants signed an informed consent form. This study was approved by the Korea Centers for Disease Control and Prevention Institutional Review Board (approval No. 2013-12Exp-03-5C).

Of the 31,207 participants in the 2014–2017 survey, we used data collected from 6653 young adults (2943 males and 3710 females) aged 20–39 years. Apparently healthy status was defined as the absence of known chronic disease, chronic kidney disease, liver cirrhosis, or cancer. Thus, we excluded individuals if they reported a chronic disease (receiving treatment or were diagnosed by a physician) including diabetes (*n* = 37), hypertension (*n* = 97), dyslipidemia (*n* = 17), coronary heart disease or stroke (*n* = 4), chronic kidney disease (*n* = 6), liver cirrhosis (*n* = 6), and cancer (*n* = 2). In addition, pregnant women (*n* = 113) were excluded. We also excluded subjects who had fasted for less than 8 h (*n* = 643) or those with missing data (*n* = 1286). Following exclusion, a total of 4442 apparently health young adults aged 20–39 years (1813 men and 2629 women) were enrolled in this study.

### 2.2. Measurement and Classification of Variables

Height was measured on a portable stadiometer and body weight was measured using a balanced scale. Body mass index (BMI) was calculated as weight (kg) divided by the height squared (m^2^). Waist circumference (WC) was measured midway between the costal margin and the iliac crest by trained research staff. Blood pressure (BP) was measured with a mercury sphygmomanometer after 5 min of rest in a sitting position and used the mean value of two separate BP measurements for analysis. After a minimum fasting time of 8 h, venous blood samples were obtained. Plasma glucose, high-density lipoprotein (HDL) cholesterol, and triglycerides were measured with a Hitachi Automatic Analyzer 7600 (Hitachi, Japan). The Hemoglobin A1c (HbA1c) level was measured using high performance liquid chromatography (Tosoh G8, Tosoh, Japan).

Self-reported questionnaires included residential area, education level, family income, smoking status, alcohol consumption, regular exercise, and total energy intake. The residential area was categorized as urban or rural based on the Korean administrative district. Regular exercise was marked as ‘yes’ when subjects regularly performed moderate exercise. The total energy intake was calculated as a sum of foods containing energy from 24 h recall nutrition survey questionnaire. 

Subjects were categorized according to glucose tolerance status [8] with normal status defined as having fasting plasma glucose (FPG) < 100 mg/dL and HbA1c ≤ 5.6%. Undiagnosed diabetes was defined as FPG ≥ 126 mg/dL or HbA1c ≥ 6.5%. Undiagnosed prediabetes was defined as 100 mg/dL ≤ FPG < 126 mg/dL or 5.6% < HbA1c < 6.5%. Undiagnosed hypertension (HTN) was defined as having systolic BP ≥ 140 mmHg or diastolic BP ≥ 90 mmHg. Undiagnosed dyslipidemia was defined as having hypertriglyceridemia (≥150 mg/dL) or low HDL cholesterol (<40 mg/dL for males and <50 mg/dL for females) [9]. For obesity measures, BMI < 25.0 kg/m^2^ was defined as normal, 25.0 kg/m^2^ ≤ BMI < 30 kg/m^2^ as obese class I, and ≥30.0 kg/m^2^ as obese class II. Abdominal obesity was defined by WC measures of ≥90 cm for males and ≥85 cm for females [10]. Subjects with a family history of diabetes were defined as those who had either a father, mother, brother, or sister with diabetes.

### 2.3. Statistical Analysis

Complex sample analysis was applied to the KNHANES data following the recommendations of the Korean Centers for Disease Control and Prevention. Continuous variables were reported as mean ± SD, and categorical variables were reported as weighted percentages. Chi-square tests were used to analyze the differences in general characteristics between groups.

Multivariate logistic regression analyses were conducted separately to evaluate associated risk factors with undiagnosed diabetes and undiagnosed prediabetes in groups stratified by sex. The covariates included in the model were age, obesity, abdominal obesity, undiagnosed hypertension and undiagnosed dyslipidemia, family history of diabetes, sociodemographic factors (residential area, family income, education), and lifestyle behaviors (smoking status, alcohol consumption, regular exercise, total energy intake). Statistical analyses were performed using SPSS (version 25.0, IBM Corporation, Armonk, NY, USA), and *p*-values less than 0.05 were considered statistically significant.

## 3. Results

The overall prevalence of undiagnosed diabetes and undiagnosed prediabetes was 1.2% (55/4442) and 25.0% (1148/4442), respectively. Across all study participants, 1.4% (29/1813) of men and 0.9% (25/2629) of women had undiagnosed diabetes. The prevalence of prediabetes was 29.1% (567/1813) in men and 20.5% (281/2629) in women. 

Table 1 and Table 2 describe the baseline characteristics across glucose tolerance status based on sex. As expected, subjects with undiagnosed diabetes or undiagnosed prediabetes were older, were more likely to be obese, had worse metabolic profiles (higher BP, higher triglyceride levels, and lower HDL cholesterol level), had a family history of diabetes, and were more likely to be current smokers compared to the healthy group in both men and women. In addition, undiagnosed HTN and undiagnosed dyslipidemia were more prevalent among those with undiagnosed prediabetes or undiagnosed diabetes, regardless of sex. Men with undiagnosed diabetes or undiagnosed prediabetes were also less likely to perform regular exercise. Women with undiagnosed diabetes or undiagnosed prediabetes were less likely to consume alcohol. There were no statistically significant subgroup differences in family income, education, area of residence, and total energy intake for both men and women. 

Table 3 shows the logistic regression analyses results for identifying risk factors associated with undiagnosed diabetes stratified by sex. Obese class II status was a significant risk factor associated with undiagnosed diabetes compared to those with normal BMI in men (odds ratio (OR): 9.808, 95% confidence interval (CI): 1.619–59.412) and women (OR: 7.719, 95% CI: 1.332–44.747). Family history of diabetes was also significantly associated with undiagnosed diabetes (OR: 3.407, 95% CI: 1.224–9.481) in women. No significant associations were found between undiagnosed diabetes and other sociodemographic factors, lifestyle behaviors, or known diabetes risk factors.

The logistic regression analyses designed to evaluate risk factors associated with undiagnosed prediabetes stratified by sex are displayed in Table 4. Increasing age (OR: 1.098, 95% CI: 1.072–1.124), obesity (obese class I OR: 1.884, 95% CI: 1.388–2.559; obese class II OR: 3.506, 95% CI: 2.019–6.089), and family history of diabetes (OR: 1.805, 95% CI: 1.382–2.357) were significant factors associated with undiagnosed prediabetes in men. Similar significant associations with undiagnosed prediabetes were found for increasing age (OR: 1.067, 95% CI: 1.045–1.089), obesity (obese class I OR: 1.708, 95% CI: 1.202–2.427; obese class II OR: 3.803, 95% CI: 1.985–7.289), and family history of diabetes (OR: 1.513, 95% CI: 1.156–1.980) in women. Women with increased alcohol consumption had a negative association (OR 0.684, 95% CI:0.539–0.867) with undiagnosed prediabetes compared to those with lower alcohol consumption. 

## 4. Discussion

This study found that obesity was significantly associated with an increased risk of undiagnosed diabetes or undiagnosed prediabetes in young adults. Family history of diabetes was only associated with risk of undiagnosed diabetes in young women. Older age and family history of diabetes were also significantly associated with undiagnosed prediabetes regardless of sex. Alcohol consumption was found to be negatively associated with risk of undiagnosed prediabetes in young women. This large population-based study aimed to explore the risk factors associated with undiagnosed glucose intolerance status in healthy young adults who were younger than 40 years. Our results suggested that targeted screening and prevention strategies among these apparently healthy individuals would result in improved overall public health and well-being in this population. 

Patients diagnosed with early-onset diabetes more rapidly develop cardiovascular risk profiles, leading to premature death, compared to those diagnosed in middle or older age [2,3,11]. In one report, during a 23-year follow-up period, older adults with diabetes diagnosed before the age of 45 years had a higher risk of CVD mortality than those with normal glucose tolerance (hazard ratio: 1.76, 95% CI: 1.04–2.98), even though the mean age of the diabetic group was 12 years less than that of the healthy group [12]. Taken together, these findings demonstrated the need for early management and prevention of early-onset diabetes.

The prevalence of undiagnosed prediabetes was 25.0% in this study. A previous study reported 28.8% of prediabetes in Chinese young adults aged <40 years [13]. Considering that our subjects are apparently healthy young adults, 25.0% of prediabetes is a relatively highly prevalence. 

Undiagnosed diabetes results in an extended period of elevated blood glucose that increases the likelihood of serious diabetic complications, particularly in younger aged adults. Identification of risk factors for undiagnosed glucose intolerance status would be valuable for targeted screening and management of diabetes and would aid in diabetes prevention. Use of these risk factors to improve screening of at-risk young adults would provide an opportunity to reduce diabetes rates and improve overall public health. 

Obesity has been well established as a risk factor for developing diabetes [14,15,16]. A previously published meta-analysis demonstrated a dose-response like relationship between BMI and type 2 diabetes, with increased BMI resulting in an 18% increase in diabetes risk [17]. In the current study, obesity status was a major risk factor of undiagnosed glucose intolerance status. Similar findings have been reported showing that obesity was more common in young adults with diabetes than in older adults with diabetes [18,19,20]. The increasing prevalence of obesity in young adults may directly contribute to the increase in diabetes prevalence in this population. Obesity is a modifiable risk factor for both diabetes and prediabetes, whereas a family history of diabetes or increased age are not. Additionally, lifestyle changes including weight control may result in an overall reduction of diabetes prevalence in young adults, particularly in those with a family history of diabetes. The improvement of eating patterns as like low-calorie and low-fat eating plan may be also helpful. 

Increasing age has also been shown to be an important risk factor for diabetes [21,22]. Based on this, younger age has been previously considered to be a relative protective factor for diabetes development. In our study, while subjects were apparently healthy at a relatively young age <40 years old, older age within the study cohort was found to be an independent factor associated with undiagnosed prediabetes. Our findings suggest that efforts should be focused on reducing diabetes risk in the high-risk, apparently healthy population, in conjunction with sustained prediabetes screening with increased age.

Family history of diabetes has also been well established as a risk factor for the development of type 2 diabetes [8,21,22]. Individuals with a family history of diabetes may share similar environments including both lifestyle and culture factors in addition to a shared genetic background. Exposure to an environment that may promote diabetes development may influence earlier age disease onset. Similar to our findings, there have been several published studies describing the association between family history of diabetes and early-onset diabetes [23,24,25]. 

Our study found that alcohol consumption was negatively associated with prediabetes; however, the possible underlying causes for these findings were unclear due to the cross-sectional nature of this study. We were also unable to obtain more specific results regarding the types of alcohol consumed, total intake duration, and average number of drinks consumed in one sitting. Several studies have suggested that moderate levels of alcohol consumption may reduce the risk for type 2 diabetes [26,27]. A more recent community-based population study showed an inverse association between alcohol consumption and diabetes risk. There were sex-based differences in the amount of alcohol that was associated with lower risk of diabetes, and the overall associations were more pronounced in participants with higher BMI [28]. Potential biological mechanisms proposed to explain the ability of alcohol to reduce type 2 diabetes risk include alcohol dependent anti-inflammatory and HDL synthesis stimulation [29,30]. In contrast, there have also been published studies which suggest that alcohol consumption has adverse effects on diabetes [31]. The overall benefits of alcohol cessation outweigh any influence alcohol consumption has on diabetes risk. Thus, our results should be carefully interpreted and further studies exploring this potential link are needed. 

The strength of this current study includes assessment of a large, population-based, nationally representative cohort with analysis of clinical, sociodemographic, lifestyle, and anthropometric measurements. However, this study did have several limitations. First, we used a cross-sectional study design that was performed across one timepoint; as a result, causal relationships could not be determined. A second limitation was the lack of an oral glucose tolerance test for identification and classification of glucose tolerance status since the study relied on a single FPG level measurement for this classification. We also were not able to classify the specific type of diabetes since C-peptide levels, genetic tests, and autoimmune markers were not examined. Our subjects were limited to apparently healthy young adults, which might cause selection bias. Lastly, we did not have data on possible changes in BMI over time, which could may have affected the study results.

## 5. Conclusions

Our findings showed that young adults were more likely to have glucose intolerance status when they were obese, when they had a family history of diabetes, and as age increased. These findings suggest a need for more precise screening, management, and prevention strategies for determining glucose intolerance status in young adults. Future directions of this work include the need for a prospective study to further investigate the relationship between the risk factors we identified and glucose intolerance status. 

## Figures and Tables

**Table 1:** Characteristics of the study population according to glucose tolerance status in men.

	Normal	Undiagnosed Prediabetes	Undiagnosed Diabetes	*p* Value
*n* (%)	1217 (69.5)	567 (29.1)	29 (1.4)	
Age (years)	28.53 ± 0.182	31.91 ± 0.283	32.45 ± 1.013	<0.001
BMI (kg/m^2^)	23.91 ± 0.110	25.81 ± 0.182	30.09 ± 1.128	<0.001
Obesity (%) ^a^				<0.001
Normal	66.7	44.7	13.1	
Obese class I	28.3	41.8	37.8	
Obese class II	5.0	13.5	49.1	
WC (cm)	82.97 ± 0.287	88.23 ± 0.474	100.59 ± 2.654	<0.001
Abdominal obesity (WC ≥ 90 cm) (%)	20.6	39.7	78.0	<0.001
SBP (mmHg)	114.12 ± 0.371	118.11 ± 0.565	124.59 ± 1.886	<0.001
DBP (mmHg)	75.46 ± 0.309	79.46 ± 0.430	85.26 ± 2.359	<0.001
Undiagnosed hypertension (%) ^b^	7.6	13.3	35.4	<0.001
HbA1c (%)	5.21 ± 0.007	5.61 ± 0.011	7.87 ± 0.426	<0.001
FPG (mg/dL)	88.98 ± 0.180	99.48 ± 0.391	171.81 ± 12.302	<0.001
TG (mg/dL)	133.21 ± 3.230	174.87 ± 5.663	350.06 ± 57.844	<0.001
HDL-C (mg/dL)	49.90 ± 0.329	46.59 ± 0.468	39.46 ± 1.438	<0.001
Undiagnosed dyslipidemia (%) ^c^	35.8	50.3	85.6	<0.001
Family history of diabetes (yes, %)	13.7	27.0	34.1	<0.001
Smoking (%)				<0.001
Never	40.5	29.8	18.8	
Past	20.7	25.0	18.0	
Current	38.8	45.2	63.3	
Alcohol drinking (%)				0.575
≤1/week	33.3	31.3	27.0	
≥2/week	66.5	68.7	73.0	
Family income ^d^ (%)				0.068
<200	15.7	11.3	18.6	
200–399	34.2	39.9	26.9	
≥400	50.1	48.8	54.4	
Less than high school education (%)	1.5	2.3	2.9	0.328
Residence in urban area (%)	54.2	48.1	47.7	0.080
Regular exercise ^e^ (yes, %)	35.3	31.1	17.4	0.049
Total energy intake (kcal)	2589.17 ± 35.660	2587.72 ± 49.098	2750.28 ± 181.685	0.679

Data are expressed as the mean ± SD for continuous variables and as weighted percentages for categorical variables. BMI, body mass index; DBP, diastolic blood pressure; FPG, fasting plasma glucose; HDL-C, high-density lipoprotein cholesterol; SBP, systolic blood pressure; TG, triglyceride; WC, waist circumference. ^a^ Obesity is defined as normal (BMI < 25 kg/m^2^), obese class I (25 kg/m^2^ ≤ BMI < 29 kg/m^2^), or obese class II (BMI ≥ 30 kg/m^2^). ^b^ Undiagnosed hypertension is defined as SBP ≥ 140 mmHg or DBP ≥ 90 mmHg. ^c^ Undiagnosed dyslipidemia is defined as triglyceride ≥ 150 mg/dL or low HDL-C (<40 mg/dL for men). ^d^ Units are in thousands of Korean won/month. ^e^ Regular exercise is indicated as ‘yes’ when the subject performs moderate exercise.

**Table 2:** Characteristics of the study population according to glucose tolerance status in women.

	Normal	Undiagnosed Prediabetes	Undiagnosed Diabetes	*p* Value
*n* (%)	2022 (78.6)	581 (20.5)	26 (0.9)	
Age (years)	29.32 ± 0.170	31.65 ± 0.279	33.80 ± 1.611	<0.001
BMI (kg/m^2^)	21.62 ± 0.079	23.44 ± 0.218	30.67 ± 1.471	<0.001
Obesity (%) ^a^				<0.001
Normal	86.7	70.3	22.6	
Obese class I	11.3	20.3	18.0	
Obese class II	2.0	9.4	59.4	
WC (cm)	72.82 ± 0.218	77.61 ± 0.530	96.05 ± 3.708	<0.001
Abdominal obesity (WC ≥ 85 cm) (%)	8.9	23.7	80.5	<0.001
SBP (mmHg)	104.43 ± 0.239	106.99 ± 0.563	114.25 ± 2.337	<0.001
DBP (mmHg)	69.67 ± 0.207	70.98 ± 0.458	77.26 ± 1.493	<0.001
Undiagnosed hypertension (%) ^b^	1.2	2.7	3.0	0.018
HbA1c (%)	5.20 ± 0.006	5.61 ± 0.013	8.12 ± 0.564	<0.001
FPG (mg/dL)	87.61 ± 0.157	96.35 ± 0.466	183.09 ± 17.739	<0.001
TG (mg/dL)	82.41 ± 1.264	100.97 ± 3.156	183.61 ± 21.010	<0.001
HDL-C (mg/dL)	58.46 ± 0.305	55.08 ± 0.526	51.27 ± 2.493	<0.001
Undiagnosed dyslipidemia (%) ^c^	27.3	39.5	73.4	<0.001
Family history of diabetes (yes, %)	18.9	27.5	60.2	<0.001
Smoking (%)				0.046
Never	82.7	83.7	69.9	
Past	10.3	8.4	6.6	
Current	7.0	7.9	23.5	
Alcohol drinking (%)				0.001
≤1/week	51.5	61.2	66.7	
≥2/week	48.5	38.8	33.3	
Family income ^d^ (%)				0.549
<200	13.4	12.2	17.6	
200–399	33.2	36.9	42.3	
≥400	53.4	50.9	40.2	
Less than high school education (%)	2.1	3.1	3.7	0.231
Residence in urban area (%)	51.7	52.5	27.0	0.129
Regular exercise ^e^ (yes, %)	27.6	25.4	30.7	0.602
Total energy intake (kcal)	1817.10 ± 19.566	1867.75 ± 34.864	1662.09 ± 164.416	0.299

Data are expressed as the mean ± SD for continuous variables and as weighted percentages for categorical variables. BMI, body mass index; DBP, diastolic blood pressure; FPG, fasting plasma glucose; HDL-C, high-density lipoprotein cholesterol; SBP, systolic blood pressure; TG, triglyceride; WC, waist circumference. ^a^ Obesity is defined as normal (BMI < 25 kg/m^2^), obese class I (25 kg/m^2^ ≤ BMI < 29 kg/m^2^), or obese class II (BMI ≥ 30 kg/m^2^). ^b^ Undiagnosed hypertension is defined as SBP ≥ 140 mmHg or DBP ≥ 90 mmHg. ^c^ Undiagnosed dyslipidemia is defined as triglyceride ≥ 150 mg/dL or low HDL-C (<50 mg/dL for women). ^d^ Units are in thousands of Korean won/month. ^e^ Regular exercise is indicated as ‘yes’ when the subject performs moderate exercise.

**Table 3:** Associated factors of undiagnosed diabetes in apparently health young adults by sex.

	Men		Women	
	Odds Ratio (95% CI)	*p* Value	Odds Ratio (95% CI)	*p* Value
Age (/1 year increasing)	1.056 (0.969–1.151)	0.211	1.044 (0.940–1.159)	0.420
Obesity ^a^				
Normal	1.00 (reference)		1.00 (reference)	
Obese class I	2.406 (0.469–12.351)	0.293	0.704 (0.128–3.881)	0.686
Obese class II	9.808 (1.619–59.412)	0.013	7.719 (1.332–44.747)	0.023
Abdominal obesity (/no) ^b^				
Yes	1.610 (0.343–7.553)	0.546	4.221 (0.896–19.882)	0.069
Undiagnosed hypertension (/no) ^c^				
Yes	1.698 (0.708–4.072)	0.235	0.227 (0.037–1.393)	0.109
Undiagnosed dyslipidemia (/no) ^d^				
Yes	3.415 (0.993–11.747)	0.051	1.700 (0.609–4.745)	0.311
Family history of diabetes				
No	1.00 (reference)		1.00 (reference)	
Yes	1.556 (0.634–3.819)	0.334	3.407 (1.224–9.481)	0.019
Smoking				
Never	1.00 (reference)		1.00 (reference)	
Past	1.104 (0.259–4.700)	0.894	0.404 (0.077–2.130)	0.285
Current	1.597 (0.528–4.836)	0.407	2.258 (0.726–7.020)	0.159
Alcohol drinking				
≤1/week	1.00 (reference)		1.00 (reference)	
≥2/week	1.119 (0.400–3.133)	0.830	0.673 (0.215–2.104)	0.496
Family income ^e^				
<200	1.198 (0.344–4.174)	0.776	1.317 (0.377–4.603)	0.666
200–399	0.679 (0.260–1.768)	0.427	1.638 (0.508–5.285)	0.409
≥400	1.00 (reference)		1.00 (reference)	
Education				
More than high school	1.00 (reference)		1.00 (reference)	
Less than high school	1.002 (0.122–8.240)	0.998	0.866 (0.117–6.405)	0.888
Residence (/urban area)				
Rural area	1.141 (0.499–2.608)	0.754	1.980 (0.738–5.310)	0.175
Regular exercise ^f^ (/yes)				
No	0.691 (0.258–1.849)	0.461	1.046 (0.395–2.769)	0.928
Total energy intake				
Low tertile	1.00 (reference)		1.00 (reference)	
Middle tertile	2.324 (0.487–11.098)	0.290	0.540 (0.175–1.667)	0.284
High tertile	2.520 (0.513–12.385)	0.255	0.565 (0.162–1.967)	0.369

CI, confidence interval. ^a^ Obesity is defined as normal (BMI < 25 kg/m^2^), obese class I (25 kg/m^2^ ≤ BMI < 29 kg/m^2^), and obese class II (BMI ≥ 30 kg/m^2^). ^b^ Abdominal obesity is defined as waist circumference ≥ 90 cm for male and ≥ 85 cm for female. ^c^ Undiagnosed hypertension is defined as SBP ≥ 140 mmHg or DBP ≥ 90 mmHg. ^d^ Undiagnosed dyslipidemia is defined as triglyceride ≥ 150 mg/dL or low HDL-C (<40 mg/dL for men and <50 mg/dL for women). ^e^ Units are in thousands of Korean won/month. ^f^ Regular exercise is indicated as ‘yes’ when the subject performs moderate exercise.

**Table 4:** Associated factors of undiagnosed prediabetes in apparently health young adults by sex.

	Men		Women	
	Odds Ratio (95% CI)	*p* Value	Odds Ratio (95% CI)	*p* Value
Age (/1 year increasing)	1.098 (1.072–1.124)	<0.001	1.067 (1.045–1.089)	<0.001
Obesity ^a^				
Normal	1.00 (reference)		1.00 (reference)	
Obese class I	1.884 (1.388–2.559)	<0.001	1.708 (1.202–2.427)	0.003
Obese class II	3.506 (2.019–6.089)	<0.001	3.803 (1.985–7.289)	<0.001
Abdominal obesity (/no) ^b^				
Yes	1.153 (0.805–1.651)	0.438	1.485 (0.977–2.256)	0.064
Undiagnosed hypertension (/no) ^c^				
Yes	0.925 (0.627–1.363)	0.692	1.232 (0.587–2.588)	0.581
Undiagnosed dyslipidemia (/no) ^d^				
Yes	1.184 (0.927–1.512)	0.175	1.209 (0.940–1.554)	0.139
Family history of diabetes				
No	1.00 (reference)		1.00 (reference)	
Yes	1.805 (1.382–2.357)	<0.001	1.513 (1.156–1.980)	0.003
Smoking				
Never	1.00 (reference)		1.00 (reference)	
Past	1.208 (0.882–1.656)	0.238	0.797 (0.549–1.158)	0.233
Current	1.112 (0.841–1.472)	0.456	1.125 (0.745–1.700)	0.575
Alcohol drinking				
≤1/week	1.00 (reference)		1.00 (reference)	
≥2/week	1.001 (0.775–1.293)	0.994	0.684 (0.539–0.867)	0.002
Family income ^e^				
<200	0.942 (0.634–1.400)	0.768	1.024 (0.673–1.557)	0.911
200–399	1.124 (0.865–1.461)	0.380	1.068 (0.834–1.369)	0.601
≥400	1.00 (reference)		1.00 (reference)	
Education				
More than high school	1.00 (reference)		1.00 (reference)	
Less than high school	1.554 (0.548–4.405)	0.497	1.118 (0.560–2.231)	0.752
Residence (/urban area)				
Rural area	1.173 (0.917–1.500)	0.203	0.838 (0.667–1.053)	0.129
Regular exercise ^f^ (/yes)				
No	0.923 (0.724–1.178)	0.521	0.981 (0.770–1.249)	0.875
Total energy intake				
Low tertile	1.00 (reference)		1.00 (reference)	
Middle tertile	1.036 (0.729–1.474)	0.842	1.112 (0.866–1.428)	0.406
High tertile	1.101 (0.799–1.517)	0.556	1.192 (0.872–1.630)	0.270

CI, confidence interval. ^a^ Obesity is defined as normal (BMI < 25 kg/m^2^), obese class I (25 kg/m^2^ ≤ BMI <29 kg/m^2^), and obese class II (BMI ≥ 30 kg/m^2^). ^b^ Abdominal obesity is defined as waist circumference ≥ 90 cm for male and ≥85 cm for female. ^c^ Undiagnosed hypertension is defined as SBP ≥ 140 mmHg or DBP ≥ 90 mmHg. ^d^ Undiagnosed dyslipidemia is defined as triglyceride ≥ 150 mg/dL or low HDL-C (<40 mg/dL for men and <50 mg/dL for women). ^e^ Units are in thousands of Korean won/month. ^f^ Regular exercise is indicated as ‘yes’ when the subject performs moderate exercise.
